# Polyglutamine Repeats Are Associated to Specific Sequence Biases That Are Conserved among Eukaryotes

**DOI:** 10.1371/journal.pone.0030824

**Published:** 2012-02-01

**Authors:** Matteo Ramazzotti, Elodie Monsellier, Choumouss Kamoun, Donatella Degl'Innocenti, Ronald Melki

**Affiliations:** 1 Dipartimento di Scienze Biochimiche, Università degli Studi di Firenze, Florence, Italy; 2 Laboratoire d'Enzymologie et de Biochimie Structurales, UPR 3082 CNRS, Gif sur Yvette, France; Universitat Autònoma de Barcelona, Spain

## Abstract

Nine human neurodegenerative diseases, including Huntington's disease and several spinocerebellar ataxia, are associated to the aggregation of proteins comprising an extended tract of consecutive glutamine residues (polyQs) once it exceeds a certain length threshold. This event is believed to be the consequence of the expansion of polyCAG codons during the replication process. This is in apparent contradiction with the fact that many polyQs-containing proteins remain soluble and are encoded by invariant genes in a number of eukaryotes. The latter suggests that polyQs expansion and/or aggregation might be counter-selected through a genetic and/or protein context. To identify this context, we designed a software that scrutinize entire proteomes in search for imperfect polyQs. The nature of residues flanking the polyQs and that of residues other than Gln within polyQs (insertions) were assessed. We discovered strong amino acid residue biases robustly associated to polyQs in the 15 eukaryotic proteomes we examined, with an over-representation of Pro, Leu and His and an under-representation of Asp, Cys and Gly amino acid residues. These biases are conserved amongst unrelated proteins and are independent of specific functional classes. Our findings suggest that specific residues have been co-selected with polyQs during evolution. We discuss the possible selective pressures responsible of the observed biases.

## Introduction

Protein aggregation is the hallmark of over 80 disorders in humans that include type II diabetes, Alzheimer's and Parkinson's diseases [1]. Nine neurodegenerative diseases are associated to the aggregation of proteins comprising an extended tract of consecutive glutamine residues within their primary structure. These diseases, termed polyglutamine-expansion diseases, include Huntington's disease (HD), several spinocerebellar ataxia (SCA) such as Machado-Joseph disease, and the spinobulbar muscular atrophy or Kennedy's disease, and are characterized by a progressive motor and cognitive degeneration that ultimately lead to death [2]–[4].

Polyglutamine-expansion diseases share a number of characteristics. The lengths of the polyglutamine repeats (polyQs) are polymorphic in the general population. The disease is triggered when the polyQ length exceeds a certain threshold. This pathological threshold varies from 20 consecutive glutamines in SCA7 to 40 or 60 in HD and SCA17, respectively [2], [3], [4]. Above the pathological threshold, the age-of-onset (AOO) of the disease, the severity of the symptoms as well as the aggregation rate of the protein *in vitro* directly correlate to the length of the polyQ [5], [6], [7]. At the genetic level the polyQs are preferentially encoded by CAG codons, rather than the codon CAA. The repetitive nature of the polyCAG homocodon is believed to be at the origin of its expansion during gene replication, leading to longer polyQs and increased severity of the disease upon gene inheritance [8], [9].

Other factors play a critical role in disease onset. Indeed, in HD, polyQ length account for only 60% of the AOO variation between individuals; this proportion is as low as 30% in the polyQ lengths twilight zone surrounding the pathological threshold [6]. The remaining variance in AOO can be attributed to environmental factors, and to genes other than HD gene [10]. The structural context of polyQs also modulates aggregation as witnessed by the aggregation of polypeptides containing 20 consecutive glutamines as in ataxin 7 while other proteins containing over 30 consecutive glutamines remain soluble. The critical role of the structural context of polyQs is further illustrated by i) the increased aggregation propensity of polyQs devoid of their flanking amino acid stretches [11] and ii) the changes in the aggregation propensity and associated toxicity upon modifications of the global protein context [12], [13], [14], [15], [16] or the polyQ adjacent regions [17], [18], [19], [20], [21], [22], [23].

Proteomes include a number of polypeptide chains with long polyQs [24] that are soluble and encoded by invariant genes. Why do the latter proteins remain soluble when other polyQ-containing proteins aggregate and cause disease? Also why are these genes invariant when other polyQ-containing proteins encoding genes exhibit significant polymorphism in polyQ lengths? Given the importance of the protein context in polyQ pathologies, we hypothesized that a specific genetic and/or protein context may have been selected to counteract the expansion of CAG repeats within genes and aggregation propensities of polyQ-containing proteins. To determine whether this is indeed the case, we identified imperfect polyQs, e.g. polyQs with insertions, in 30 eukaryotic, bacterial and archaeal proteomes and analyzed the sequence characteristics of these proteins. Both the nature of residues flanking the polyQs and, for the first time, that of residues other than Gln within polyQs (insertions) were assessed systematically. We discovered strong amino acid residues biases robustly associated to polyQs in all the eukaryotic proteomes we examined, with an over-representation of Pro, Leu and His and an under-representation of Asp, Cys and Gly amino acid residues. These biases are conserved amongst unrelated proteins and are independent of specific functional classes. Our findings suggest that specific residues have been co-selected with polyQs during evolution. The selective pressures responsible of the observed biases and the potential origin of the polymorphism in polyQ lengths are discussed.

## Results

### A census of Human imperfect polyQ stretches

**Figure 1:**
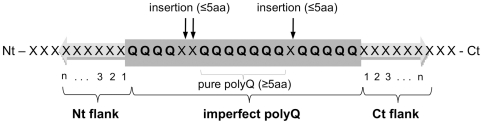
Schematic of a polyQ. A polyQ contains a minimum of five consecutive Q residues. The maximum proportion of residues other than Q (insertions) is 25% and each insertion cannot be over 5-residues long. The N- and C-terminal flanks of the polyQ are labeled Nt and Ct flanks, respectively. The numbering scheme for the residues within the flanks is shown.

We developed a computer program designed to scrutinize entire proteomes for polyQ stretches and their corresponding coding sequences (see Methods). The insertions of various amino acid residues within the polyQs was allowed given that polyQs are imperfect in a number of polyQ-expansion diseases as exemplified by the two His insertions in the middle of ataxin 1 polyQ in most healthy individuals that are systematically absent in individuals developing SCA1 [25]. PolyQs were defined as tracts with a minimum Q content of over 75%, and a core of at least five consecutive Q that can be extended N- and/or C-terminally with insertions of non Q residues of no more than 5 consecutive residues (Figure 1). The rationales for these cut-offs are the followings. For a protein of average length and residue composition, a cut-off of 5 consecutive residues allows to retrieve only statistically significant stretches [26]. Similarly, a minimum Q content of 75% provides enough inserted residues to allow statistical analyses. Thus defined, polyQs may have variable lengths (with a minimum of 5-residues) and amino acid composition (Q ranging from 75% to 100% of the residues). In addition, any protein can contain more than one polyQ. The engine was also designed to retrieve the primary structures flanking polyQs, which were denominated N-terminal (Nt) and C-terminal (Ct) flanks, respectively. Finally, the engine associates to each polyQ stretch the identity and function of the corresponding protein e.g. Description (from the EnsEMBL database), Gene Ontology (GO) and Online Mendelian Inheritance in Man (OMIM). To improve the search engine sensitivity and avoid redundancy, alternative transcripts as well as proteins tagged as “hypothetical” or devoid of annotations were not considered. It is worth noting that the analytical tools developed for this work are flexible and can be used to retrieve any kind of imperfect polyX repeat.

**Figure 2:**
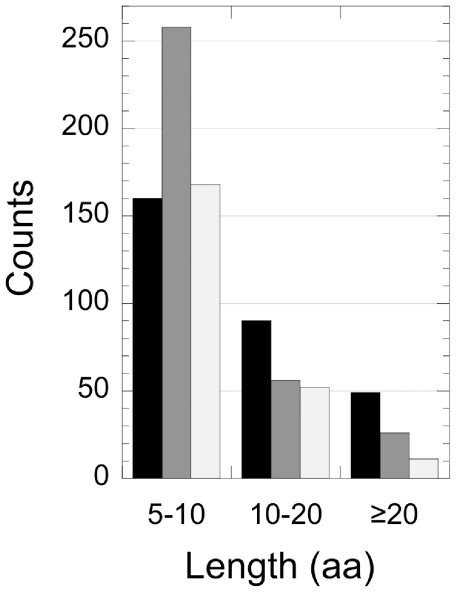
Size distribution of the polyQs in the human proteome. Black, imperfect polyQs; dark gray, pure polyQs; light gray, pure polyCAGs.

We applied the search engine we designed to the human proteome and retrieved 299 polyQs, distributed within 248 proteins. 240 proteins contained 1 or 2 polyQs while only 8 contained 3 polyQs or more. The 9 polypeptides involved in polyQ-expansion diseases were correctly retrieved. The polyQ lengths spanned from 5 to 100 residues. 49 stretches (16%) were over 20 residues long (Figure 2). The ability of our search engine to accommodate insertions allowed us to retrieve as single polyQs a number of sequences that would have been identified otherwise as distinct hits (Figure 2). The whole human output is available at the AAstrech Project website www.unifi.it/scibio/bioinfo/aastretch/.

### PolyQs are associated to diseases in humans other than the canonic expansion diseases

We first analyzed the OMIM records associated to human polyQs. Significant associations between OMIM tags and pure polyX stretches have been previously reported [27]–[29]. We confirmed this result by showing that 37 out of the 248 polyQ-containing proteins had an OMIM record. This proportion (15%) was significantly higher than that of the OMIM-tagged proteins in the input database after removal of the hypothetical/not-annotated entries (10%; *P*<0.05; Fisher's exact test).

**Figure 3:**
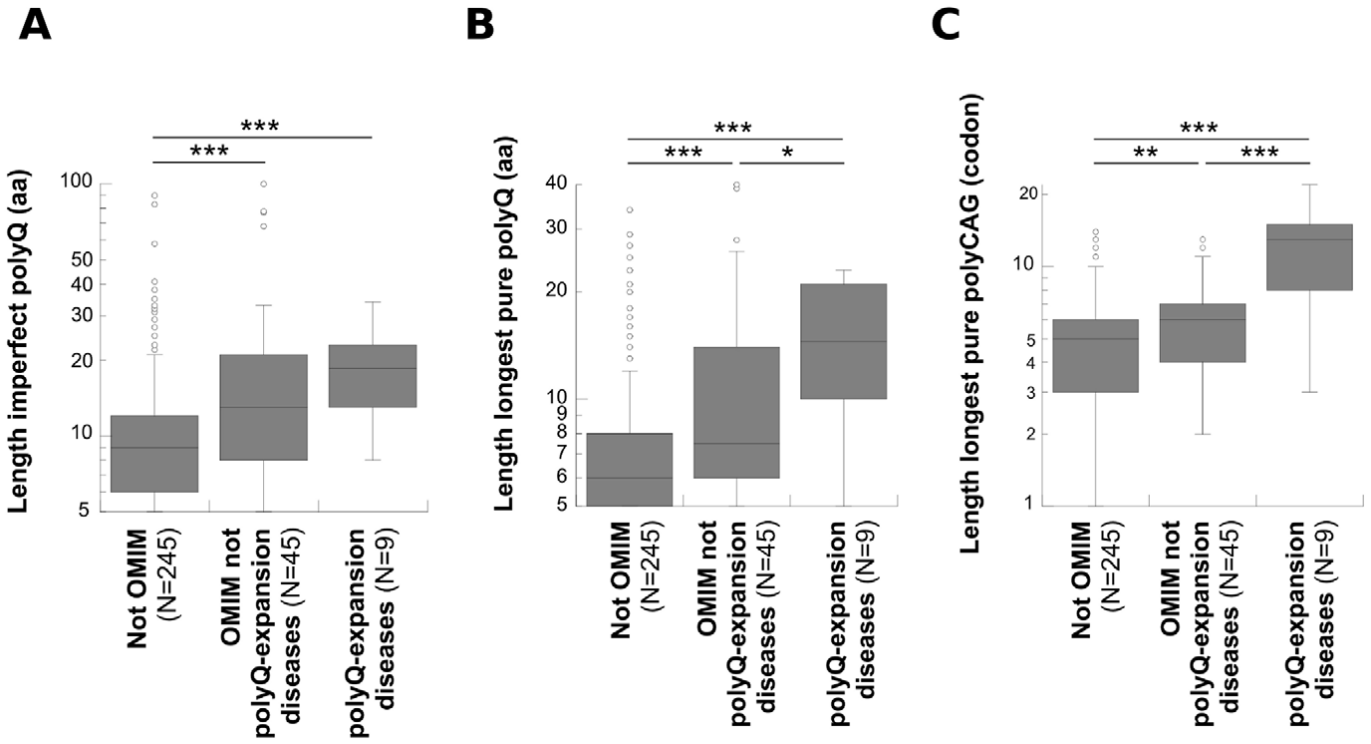
PolyQs associated to human diseases are significantly longer than those that are not. (A) Length of the imperfect polyQs. (B) Length of the longest pure polyQ within each polyQ zone. (C) Length of the longest pure polyCAG within each polyQ zone. ***, p<0.001; **, p<0.01; *, p<0.05 (Mann-Whitney and Kolmogorov-Smirnov tests).

To further analyze the link between polyQ-containing proteins and genetic diseases, we organized human polyQs into three classes: polyQ-expansion diseases (N = 9), OMIM-tagged polypeptides that are not associated to polyQ-expansion diseases (N = 45), and OMIM-untagged polypeptides (N = 245). We then compared the length of polyQs in these three classes (Figure 3). As expected, we found the polyQs involved in expansion diseases the longest (Figure 3A), the richest in pure polyQ repeats (Figure 3B) and encoded by the longest pure polyCAG homocodons (Figure 3C). Interestingly, polyQs associated to other genetic diseases were also longer (Figure 3A; *P*<0.001), with higher pure polyQ content (Figure 3B; *P*<0.001) and encoded by longer polyCAGs (Figure 3C; *P*<0.01) than disease-unrelated polyQs. This observation establishes a link between proteins with long polyQs and human genetic diseases other than the canonic expansion diseases. Noticeably, polyQs polymorphism is also associated to other human diseases. Indeed, we screened the literature for the terms “polyglutamine” and “polymorphism”, and identified several human multifactorial diseases for which an allele of specific polyQ length is a risk factor (Table S1). For example, somatic polymorphism in AIB1 polyQ length is a genetic risk factor that influences breast cancer onset [30], [31], while a polyQ longer than 40 or shorter than 22 residues in the androgen receptor is associated to the expansion disease spinobulbar muscular atrophy or constitute a risk factor for the prostate cancer, respectively [32].

### Sequence biases associated to human polyQs

To determine whether particular residues are associated specifically to human polyQs, we calculated the sequence biases associated to polyQs as detailed in the Methods section. Briefly, we first calculated the occurrence of each of the 19 amino acid residues within polyQ insertions. The occurrence of each residue was then expressed as a fraction of the total number of residues inserted within a polyQ (*F*_insertion_). The frequency of each amino acid residue within the insertions (*F*_insertion_) was then expressed as a function of its mean frequency of occurrence within the whole proteome (*F*_proteome_), so that residues of different natural abundances can be readily compared. A residue was considered over-represented when *F*_insertion_/*F*_proteome_ was >2, i.e. the residue was twice as frequent within polyQ insertions as compared to the proteome. Under-represented residues had an *F*_insertion_/*F*_proteome_<0.5, i.e. the residue was twice less frequent within polyQ insertions as compared to the proteome.

**Figure 4:**
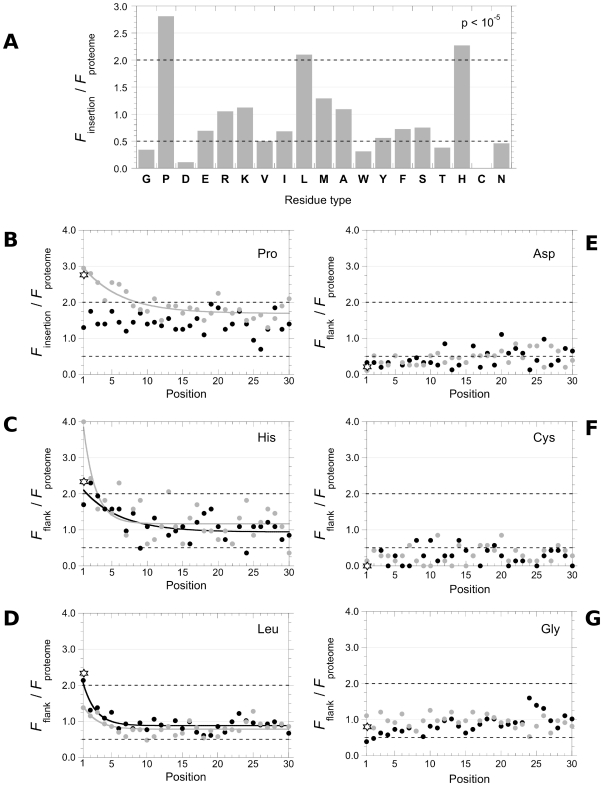
Sequence biases associated to polyQs in the human proteome. (A) Sequence biases within polyQs (insertions). The relative abundances of each residue type within polyQs are represented. (B–G) Sequences biases at the flanks of polyQs. Each point represents the relative abundance of Pro (B), His (C), Leu (D), Asp (E), Cys (F) and Gly (G) at each of the 30 positions within the Nt (black circles) and Ct (gray circles) flanks. The relative abundances within the polyQ insertions are also indicated (stars). The solid lines are the best fit to an exponential function. The dotted lines indicate the threshold for residues over- (residue twice as frequent as in the proteome) or under- (residue twice less frequent than in the proteome) representation.

The results of our analysis are presented in Figure 4A. The overall distribution of the 19 residues within polyQs was significantly different from that in the whole human proteome (Figure 4A; *P*<10^−5^; Chi-square test) and in the polyQ-containing proteins (not shown; *P*<10^−5^; Chi-square test). These results reveal the existence of residue biases within polyQ insertions. Three residues were over-represented: Pro, Leu and His, while four residues were under-represented: Asp, Cys, Gly and Trp. As Trp is particularly rare in the genomes and given that no bias for aromatic residues taken as a whole is observed, Trp bias was not considered any further.

The flanks of human polyQs were subjected to the same analysis (Figure 4B–G). The relative abundance of each residue at each position j within the Nt and Ct flanks was calculated (*F*_flank, j_/*F*_proteome_). The amino acid position j was defined as 1 for residues flanking the polyQ stretch on the N- and C-terminal sides, the preceding residue on the N-terminal and following residue on the C-terminal sides were numbered 2, etc… (see Figure 1). Flank analyses were limited to 30 residues N- and C-terminally to the polyQ. At further positions, the different flanks cannot be easily aligned, and the analyses will depend on the intrinsic aggregation propensities of the stretches considered. A bias in residue distribution was observed for the residues adjacent to the polyQs with an over-representation of Pro, His and Leu (Figure 4B–D) and an under-representation of Asp, Cys and Gly (Figure 4E–G). The *F*_flank,1_/*F*_proteome_ values of the two flanks were quantitatively similar to that of *F*_insertion_/*F*_proteome_ (stars in Figure 4B–G). The relative abundances of these residues reached the standard distribution (*F*_flank,j_/*F*_proteome_ = 1) when the distance between the residue and the polyQ increased. With the exception of Pro at the Nt flanks, best fits to linear regressions over the first ten positions of both Nt and Ct flanks were statistically significant, (*P*<0.01 in all cases; result not shown), further demonstrating the significance of the bias we report. The two flanks were globally symmetrical, displaying similar residue compositions. Pro distribution within the two flanks was asymmetric: this residue was over-represented only within the Ct flanks (Figure 4B), and often as a polyP ranging from 3 to 11 consecutive Pro (22% of the sequences we examined). PolyH were also observed while Leu residues were almost never found clustered but rather regularly spaced.

**Figure 5:**
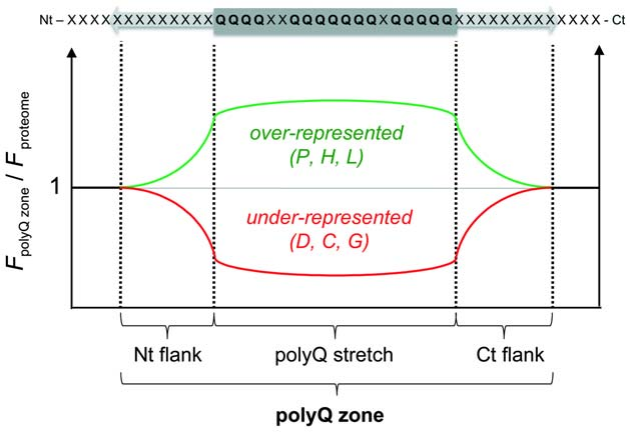
The polyQ zone. PolyQs insertions and flanks share identical residue biases: Pro, Leu and His are over-represented within these zones while Asp, Cys and Gly are under-represented.

In summary, sequence biases were observed for the same residues both within and at the flanks of polyQs. Thus, polyQs appear to be organized into regions (hereinafter termed polyQ zones) with a peculiar composition where Pro, His and Leu residues are enriched and Asp, Cys and Gly selectively excluded (Figure 5).

### The sequence biases associated to polyQs are conserved throughout eukaryotes

To determine whether the sequence biases associated to polyQs we identified are specific to humans or conserved across living organisms, 16 eukaryotic, 8 bacterial and 5 archaeal proteomes, listed in Table 1, were subjected to the same analysis performed on human proteome and described above. We chose proteomes which annotation was of adequate quality and exemplifying organisms that sampled to the best the diversity of living organisms. As previously reported for pure polyX stretches [27], [33], bacterial and archaeal proteomes were devoid of polyQs. The numbers of polyQs and polyQ-containing proteins in each of the eukaryotic proteomes we examined are listed in Table 1. The 15 eukaryotic proteomes containing over 100 polyQs were further examined. The corresponding outputs are available at the AAstrech Project website www.unifi.it/scibio/bioinfo/aastretch/.

**Table 1:** Amount of polyQs in eukaryotes.

Organism	Group	N entries	N polyQs	N polyQ proteins
Human (*Homo sapiens*)	primate	37 067	299	248
Chimpanzee (*Pan troglodytes*)	primate	27 903	227	193
Orangutan (*Pongo pygmaeus*)	primate	19 440	236	189
Mouse (*Mus musculus*)	rodent	32 698	314	262
Dog (*Canis familiaris*)	mammal	20 037	170	135
Xenopus (*Xenopus tropicalis*)	amphibian	15 971	133	117
Chicken (*Gallus gallus*)	bird	15 177	165	135
Zebrafish (*Danio rerio*)	fish	17 275	191	159
Fruit fly (*Drosophila melanogaster*)	insect	18 253	2 242	1 162
Worm (*Caenorhabditis elegans*)	nematode	20 878	259	192
Fungus (*Aspergillus niger*)	ascomycota	11 746	159	137
Yeast (*Saccharomyces cerevisiae*)	ascomycota	6 416	187	141
Yeast (*Schizosaccharomyces pombe*)	ascomycota	4 958	19	16
Amoeba (*Dictyostelium discoideum*)	protist	5 856	1 792	1 005
Plasmodium (*Plasmodium falciparum*)	protist	5 331	27	27
Rice (*Oryza sativa*)	angiosperm	52 694	869	789
Thale cress (*Arabidopsis thaliana)*	angiosperm	31 344	340	285

The search engine was applied to 17 eukaryotic proteomes in search for imperfect polyQ repeats. N entries refer to the total number of proteins present in the input proteome after isoforms removal (see Methods); N polyQs refer to the number of imperfect polyQ repeats retrieved by the program (see Methods and Figure 1 for definition of an imperfect polyQ repeat); N polyQ proteins refer to the number of polyQs-containing proteins.

**Figure 6:**
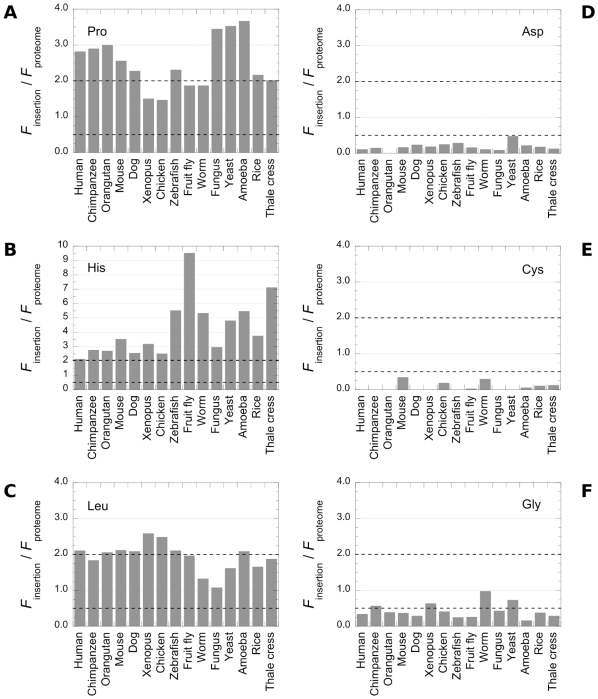
Sequence biases associated to polyQs are conserved throughout eukaryotic proteomes. The relative abundances of Pro (A), His (B), Leu (C), Asp (D), Cys (E) and Gly (F) within polyQs insertions in the 15 eukaryotic proteomes we analyzed are represented. The dotted lines indicate the threshold for residues over- (residue twice as frequent as in the proteome) or under- (residue twice less frequent than in the proteome) representation.

Pro, His and Leu were found over-represented (Figure 6A–C) while Asp, Cys and Gly under-represented (Figure 6D–F) within the polyQs throughout the proteomes we analyzed, with very few exceptions. No other residues exhibited biases. The biases in some cases were more obvious than in humans. His residue was for example 10-times more frequent within polyQ insertions in fruit fly, representing almost ¼ of the insertions (Figure 4B). A strong bias for His was also observed in zebrafish, worm, yeast, amoeba and thale cress (Figure 4B). The amino acid biases at the Nt and Ct flanks (examples are presented in Figure S1[see additional data 1]) were assessed. The same biases we report in human were observed in the different proteomes. Interestingly, the asymmetrical repartition of Pro residues appears to be a recent outcome, as it was not observed for all the organisms (Figure S1A). We conclude from our observations that Pro, His and Leu are associated to, while Asp, Cys and Gly are excluded from the polyQ zones in the 15 eukaryotic proteomes we analyzed.

**Figure 7:**
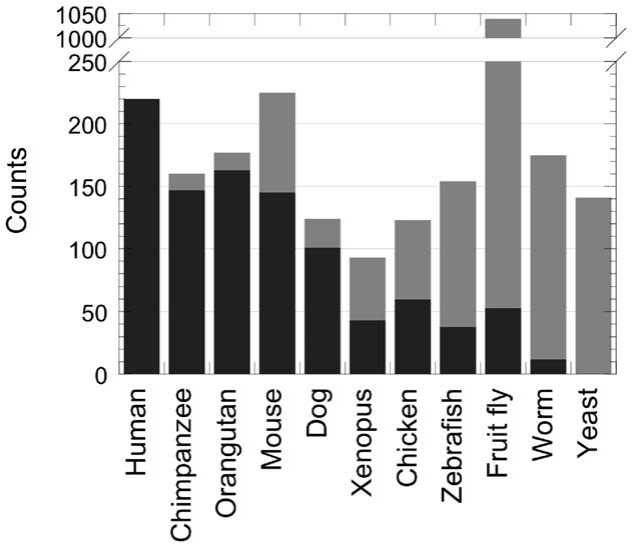
Eukaryotic polyQ-containing proteins are not orthologous. The number of polyQ-containing proteins that are orthologous to human polyQ-containing proteins in different organisms are highlighted in black. Those that are not are in gray.

To ascertain that the strong conservation we observed is not the consequence of a common evolutionary origin of the proteins we analyzed, we mapped the orthology relationships of human polyQ-containing proteins in a panel of organisms (see Methods; Figure 7). In mammals a significant fraction of polyQ-containing proteins had orthologs among human polyQ-containing proteins (Figure 7). This proportion decreased dramatically for non-mammalians, with only 5% of the fruit fly and none of the yeast polyQ-containing proteins having orthologous counterparts among human polyQ-containing proteins (Figure 7). Thus, the sequence biases associated to polyQs we observed within the 15 eukaryotic proteomes we analyzed are not due to common evolutionary origin of the polyQ-containing proteins. To confirm this result, we removed proteins orthologous to human polyQ-containing proteins from non-human proteomes. The same sequence biases within polyQs insertions (Figure S2 ) and at their flanks (not shown) occurring at similar frequencies were observed for the 15 eukaryotic proteomes lacking or not proteins orthologous to human polyQ-containing proteins.

### Codon usage

It has been reported that homocodon repeats are often interrupted by single base-pair mutations [34], [35]. To determine whether the residue biases associated to polyQs are the consequence of single base-pair mutations affecting polyCAG homocodons, we analyzed the codons preferentially used in mammals to encode the amino acid residues over-represented within the polyQ insertions.

**Figure 8:**
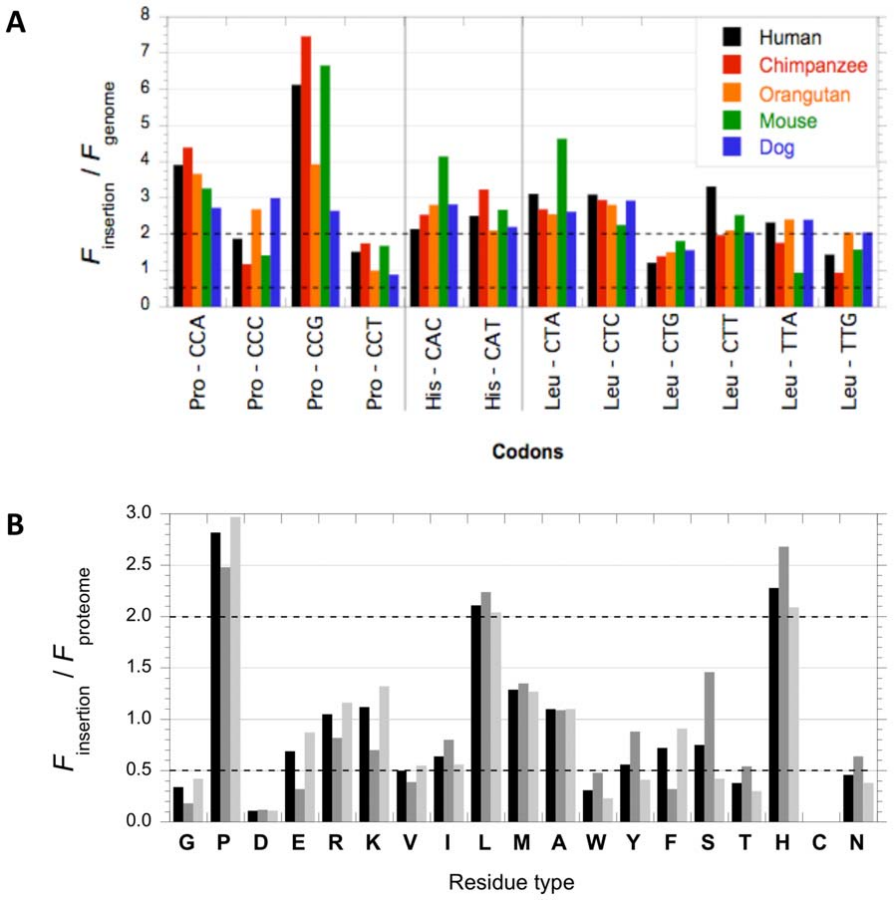
A survey of additional biases within polyQs. (**A**) **Codon biases within polyQ insertions**. The relative abundances of the different Pro, His and Leu codons within polyQs insertions in human, chimpanzee, orangutan, mouse and dog are represented. The dotted lines indicate the threshold for codons over- (codons twice as frequent as in the rest of the open reading frames) or under- (codons twice less frequent than in the rest of the open reading frames) representation. (**B**) **Sequence biases within human polyQs are not specific to transcription factors**. The relative abundance of each residue within polyQs is represented for all human polyQs (black), for human polyQs tagged by the GO term “regulation of transcription DNA-dependent” (dark gray) and human polyQs that are not tagged by the GO term “regulation of transcription DNA-dependent” (light gray). The dotted lines indicate the threshold for residues over- (residue twice as frequent as in the proteome) or under- (residue twice less frequent than in the proteome) representation.

The six codons for Leu were equally represented (Figure 8A). The codon CTG is the only Leu codon that can result from a single base-pair mutation within the CAG codon. Its occurrence is however the lowest. A strong bias for 2 codons out of 4 encoding Pro (CCG and CCA) within polyQ zones was observed. Both are one mutational event apart from the CAG and CAA codons that encode Gln. Interestingly, a CCG codon was significantly more often found flanking a sequence ending with a CAG as compared to similar sequences ending with a CAA (*P* = 0.003; Fisher's exact test). This suggests that the CCG codon arises from CAG codons. The two His codons (CAC and CAT) are one mutational event apart from the CAG codon. Both were equally over-represented. Similar biases were found at polyQs flanks. We conclude from these observations that Pro and His but not Leu residue over-representations could arises from single mutational events within polyCAG codons.

### Functional specificities associated to polyQs do not account for the observed sequence biases

Specific functional classes have been reported to be over-represented within polyQ-containing proteins, in particular, transcription regulatory proteins [36]–[39]. This could account for the observed sequence biases. Indeed, transcription factors DNA-binding domains can be enriched in basic residues and impoverished in acidic residues [40], [41]. Thus, the biases we observe for His and Asp could be due to a convergent evolution of transcription factors.

Almost half of the GO tags “Biological process” associated to human polyQ-containing proteins are labeled “regulation of transcription DNA-dependent” (GO0006355; Figure S3A–C), thus confirming the strong representation of transcription factors within this protein category. However, the sequence biases we observed (over-representation of Pro, His and Leu and under-representation of Asp, Cys and Gly) were indistinguishable when we analyzed proteins tagged “regulation of transcription DNA-dependent” or not (Figure 8B). We also analyzed the GO tags associated to polyQs in the other eukaryotic proteomes we selected. In a number of cases a majority of the polyQ-containing proteins were found tagged “regulation of transcription DNA-dependent”. However, this was not the case for fruit fly, worm, amoeba and rice, where this class of proteins represents less than 10% of the GO-tagged polyQ-containing proteins (Figure S3D). We conclude from these observations that the sequence biases associated to polyQs in eukaryotes are not specifically associated to transcription regulating proteins.

## Discussion

### PolyQ zones

We assessed the sequence biases associated to imperfect polyQ repeats in 15 different eukaryotic proteomes. We discovered that Pro, His and Leu are specifically over-represented both within insertions inter-spreading tracts of pure polyQ and within the 10–15 first positions of the sequence immediately preceding (Nt flank) or following (Ct flank) the polyQ as compared to their distribution within the whole proteome. In contrast Asp, Cys and Gly are specifically excluded from these regions. PolyQs appear therefore associated to other sequence characteristics, thus defining polyQ zone: where Q repeats are flanked by Pro, His and Leu rich amino acid stretches that lack specifically Asp, Cys and Gly residues. As previously reported [24], [36]–[39], these very specific sequence tracts were not randomly distributed in the proteomes but embedded in proteins with different functions within a given organism. This observation confirms the existence of a positive functional selection acting on polyQs [42].

### Origin of polyQs, their flanks and insertions

We observed the same residue biases that define polyQ zones in 15 different proteomes sampling a wide diversity of eukaryotic classes, displaying different genome sizes and different abundances of polyQs (Table 1). Remarkably, these robust biases are not the consequence of a conservative evolution from common protein ancestors (Figures 7 and S2). Indeed, if many of the human polyQ-containing proteins orthologues are conserved amongst eukaryotes, polyQ presence and length within a given protein are rarely conserved [24], [27], [29], [35]–[37], [43], [44]. Huntingtin for example is conserved in all deuterostomia; however, only vertebrate's huntingtin contains polyQs and they are of different lengths [45]. Given the limited polyQs conservation level amongst orthologous proteins, the robust sequence biases associated to polyQs in unrelated proteins is noteworthy.

We can not exclude that polyQs may have originated from mutational events within amino acids repeats codons which traces remain tightly associated to their flanks. This could be particularly true for Pro and His residues which codons are one mutational event from the CAG codon and that are found as homopeptidic repeats. Several lines of evidence argue however against this view. First, the flanking polyP and polyH were always encoded by mixed codons (not shown). Second, polyQs in drosophila are not encoded by polyCAGs but rather by mixed CAG and CAA codons [46] that are unlikely to originate from mutational events within polyP, mostly encoded by CCG codons. Nonetheless, they are associated to the same residue biases. Finally, these residues often appear after the polyQs during evolution [47]. For example, the polyP flanking huntingtin's polyQ is found only in mammals not in other vertebrates [45]. Similarly, the His insertions that is lacking in Old World monkeys ataxin 1 appeared later on, during great apes evolution [48].

Thus, the polyQ-associated sequence biases appear to be the consequence of a strong selection pressure at the molecular level. One can hypothesize that the biases confer a selective advantage to the polyQ-containing genes and proteins. The nature of this advantage is however difficult to identify. Different non-exclusive selection pressures could be at the origin of the observed biases. The current knowledge arguing for or against each of them are discussed hereinafter.

### Selection pressures exerted on polyQ zones

#### Genetic stability of polyCAGs

The global genomic context in which a polyQ stretch is embedded impacts the genetic stability of the repeat, i.e. its propensity to expand or not during replication [49], [51], [52]. In particular, homocodon interruptions improve genetic stability. The human ataxin 1 allele displaying a 39-CAG tract with a single CAT (His) interruption is for example stable, whereas alleles that have a pure repeat of the same length are not [8], [50]. The mechanisms underlying genetic stabilization are not clear. Codon interruptions could inhibit the slippage between nascent and template strands during replication, thus keeping them in register, and/or destabilize the hairpin structures formed by the slipped DNA strands [8], [53]. Thus, single mutational events within or at the flanks of polyCAGs may be directed toward increasing their genetic stability.

#### Toxicity of aberrant mRNA structures

A polyCAG transcripts-associated toxicity has been reported in three different model systems [54]–[56]. The molecular basis of this toxicity is not fully understood. Pure polyCAG RNAs as well as transcripts of polyQ-containing proteins can form both *in vitro* and *in vivo* stable double-stranded (dsRNA) hairpins, in which non-canonical A-A base pairs are stabilized at each side by 2 consecutive C≡G and G≡C base pairs [57]–[59]. These dsRNA structures could specifically bind dsRNA-binding proteins, modifying their availability, thus, eliciting toxicity [60]. Alternatively, these dsRNA may be cleaved into siRNA and target the expression of other genes with complementary sequences [61]. PolyCAG flanks are believed to be involved in the hairpin formation and stabilization [57], [62], [63]. Interruption of the polyCAG by a CAT and to a lesser extent a CAA codon in the SCA1 and SCA2 transcripts, respectively, destabilizes the hairpin structures [64]. Furthermore, a single CAA insertion within an untranslated polyCAG dramatically reduces polyCAG-associated toxicity in a fruit fly model [55]. Thus, single mutational events within or at the flanks of polyCAG codons may be directed toward reducing dsRNA toxicity.

Nonetheless, the most favoured mutations to interrupt a polyCAG tract are theoretically single base pair mutations, and in the first place the synonymous and transition mutation leading to a CAA (Gln) codon. Other single base-pair mutations lead to the insertion of a His (CAT or CAC), a Lys (AAG), a Glu (GAG), a Leu (CTG), a Pro (CCG), an Arg (CGG) or a stop codon (TAG). The fact that non-synonymous mutations are found within polyQ zones, and that codons CAT, CAC and CCG encoding His and Pro are preferred over other codons suggests the existence of additional selection pressures at the protein level.

#### PolyQ-containing proteins aggregation and function

PolyQs length modulates the aggregation propensity of polyQ-containing proteins. The resulting protein aggregates are toxic to the cell constituting a prime component of the pathology in polyQ expansion diseases [2], [65]. A number of experimental reports suggest that some of the sequence biases we observe within polyQ zones interfere with protein aggregation. In a yeast model expressing huntingtin exon 1, polyP deletion increases the number and toxicity of cellular inclusions [19], [20], [66]. Furthermore, *in vitro*, polyP stretches affect the aggregation of polyQ-containing polypeptides [17], [18], [22], [67], [68]. It seems reasonable to attribute these effects to the conformational rigidity and structure breaker properties of Pro. PolyPro are also expected to fold into PPII helices that constrain the adjacent polyQ tract in a PPII-like and non-aggregation prone structure [18], [69]. Similarly His insertions increase the solubility and decrease the aggregation rates of polyQ containing polypeptides *in vitro*, in addition to modifying the structure of the resulting aggregates [68], [70]–[71]. The main hypothesis is that the imidazole rings favour intra- over inter-molecular hydrogen bonds, thus acting as a ß-sheet breaker [68]. His residues role could also depend on the environment, in particular their protonation state [70]. Thus, the sequence biases we observe within polyQ zones, in particular the presence of Pro and His, may have been selected for to disfavour polyQ-containing protein aggregation. This “gatekeeper” function would be analogous to the one attributed by Rousseau et al. to the residues over-represented at the flanks of amyloid-forming polypeptide stretches [72]. However, it should be noted that the nature of the residues used as gatekeepers is different in the two cases, highlighting the peculiarity of polyQ-protein aggregation.

The rationale for the over-representation of Leu and the under-representation of Asp, Cys and Gly we observed is unclear as opposed to that of Pro and His. Cys residues are highly reactive and as such, may be disadvantageous in a particular structural context. The same reasoning cannot be applied to the other residues. Their over- or under-representation remains therefore intriguing. We cannot exclude that the biases we observe are the consequence of a convergent evolution for functional requirements. However, we believe such functional bias unlikely, as the residue biases we observe do not depend on any functional specificity, in particular the overrepresentation of transcription factors amongst polyQ-containing proteins. Further annotation improvements and identification of protein functions will strengthen or weaken our beliefs.

### PolyQ-containing proteins, gatekeepers and human diseases

PolyQs together with the specific residue biases they are associated to define polyQ zones in eukaryotes. These zones could originate from different and non-exclusive selection pressures, both at the genetic and protein levels, that could account for the restricted expandability and aggregation propensity of the hundreds of polyQ-containing proteins present within the eukaryotic proteomes. The fine tuning of polyQ-protein sequences we observe is probably the result of a trade-off between the functional requirement for polyQ stretches, mainly involved in protein-protein interactions and transcriptional regulation [27], [29], and their potential noxiousness. We can speculate that mutations of the gatekeeper residues within polyQ zones could perturb this delicate balance and trigger new disorders, as previously observed for amino acid stretches that are prone to form amyloids [73]. Our results further highlight the role of the different selection pressures exerted by protein aggregation on shaping proteomes sequences [74].

## Methods

### Data collection

Human and other eukaryotic proteomes and transcriptomes, as well as annotations, Gene Ontology (GO) [75] terms, OMIM codes [76] and ortholog coordinates were obtained from EnsEMBL [77], [78] in October 2010. As our engines (*AAstretch* and *AAsync*) requires a file with a predefined dataset, we set up a script (*AAprepare*) to automatically download and prepare the input files using EnsEMBL ftp site and BioMart [79]. For each organism the input structure consists of two fasta-formatted files, one containing open reading frames, the other the corresponding protein sequences. The integrity, accuracy and correspondence of the open reading frames and the proteins they encode were checked through an automatized procedure followed by random manual control. The corresponding sequences in the two files had exactly the same fasta header containing the following items as vertical bar-separated and ordered fields: the protein code, the transcript code, the gene code, the transcript description, the GO function, the GO biological process, the GO cellular component and, for human proteins, the OMIM annotations. This automated approach allowed us to generate input files for most of the organisms available at www.ensembl.org and at www.ensemblgenomes.org. Bacterial (*E. coli*, *B. subtilis*, *M. tuberculosis*, *A. variabilis*, *F. psychrophilum*, *B. burgdorferi*, *C. aggregans* and *T. maritima*) and archaeal (*S. solfataricus*, *M. voltae*, *M. jannaschii*, *M. smithii* and *Holobacterium sp.*) proteomes were obtained from the NCBI database. In that case the COG code [80] was associated to each protein/gene. Orthology relationships between organisms were extracted from the EnsEMBL biomart repository. Codon usage tables were reconstructed directly from the input files prepared by *AAprepare*. The data processing we used is schematized Figure S4.

### Search engines

The core search engines we developed for this work are described in depth in the manual available at the AAstrech Project website www.unifi.it/scibio/bioinfo/aastretch/. Briefly, the program *AAstretch* analyzes files generated by *AAprepare*. It retrieves within every sequence in the proteome all the imperfect polyX stretches (polyXs) as defined in Figure 1, X being any residue. The engine locates within a given protein sequence a pure polyX of a minimal length that is defined a priori. It then tries to extend N- and C-terminally the match tolerating the insertion of residues other than X. Each insertion cannot be longer than a defined length and the overall proportion of residues other than X within the polyX is defined as well. The proteome, the identity of the residue constituting the polyX, the minimal length of pure polyX, the maximal length of insertions and the minimal proportion of residue X within the polyX are defined by the user. The AAsync program generates a polyX and flanks open reading frame file from the AAstretch output file. X was set to Q within this work. We will refer hereinafter to polyQs.

The *AAstretch* output is a tab-delimited file that associates to each polyQ: 1- its sequence, 2- its length, 3- the sequences of its N- and C-terminal flanks, 4- the proportion of Q residues within the polyQ, 5- the location of the polyQ within the protein, 6- the length of the longest pure polyQ within the stretch and 7- the protein GO and OMIM annotations. The datasets can be filtered to reduce redundancy and improve accuracy. This analysis scheme allows keeping within the dataset only one polyQ for alternative transcripts harboring identical stretches and flanks. We used the EnsEMBL coding system for “alternative transcripts” definition, which considers alternative transcripts of a gene those entries marked by different transcript and protein codes though sharing the same gene code. Another filtering scheme allows to remove entries with descriptions fields matching a given term. In the present analysis, entries devoid of annotations and labeled “hypothetical” were removed from the dataset.

Other options available but not used in this work are described in details in the manual. In particular, *AAstretch* can retrieve X-enriched areas, in which the minimal proportion of X can be considerably lower than that set throughout this work, with no minimal pure polyX length.

### Analysis tools

We designed an interactive graphic tool, *AAexplore*, that represents automatically the *AAstretch* output files in a readily interpretable form supported by chi-squared statistics and plots.

*AAexplore* calculates the polyQs insertions sequence bias for each of the 19 amino acids other than Q. The frequency at which X is found within the polyQ (*F*(X)_insertion_) is first calculated using the equation:
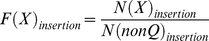
(1)where *N*(X)_insertion_ is the occurrence of X and *N*(nonQ)_insertion_ the occurrence of all residues other than Q (including X) within polyQ insertions in all the output.

Then the frequency at which X is found in the background (*F*(X)_background_), is calculated:
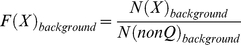
(2)where *N*(X)_background_ is the occurrence of X and *N*(nonQ)_background_ the occurrence of all residues other than Q within the background. Two different backgrounds can be used, namely the whole proteome or the proteins that constitute the output files. Finally *AAexplore* computes a bias function (*Bias*(X)_insertion_) so that residues of different natural abundances can be compared:
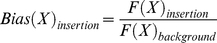
(3)Similarly, the sequence bias at each position j of the flanks (*Bias*(X)_flank, j_) for each of the 19 amino acids other than Q is calculated using equations (4–5):
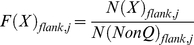
(4)

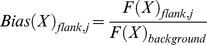
(5)where *N*(X)_flank,j_ is the occurrence of X and *N*(nonQ)_insertion,j_ the occurrence of all residues other than Q at position j within all polyQ flanks.

*AAexplore* displays a bar plot summarizing *N*(X), *F*(X) or *Bias*(X) for the insertions, the Nt flank, the Ct flank or the two flanks taken together for each amino acid. Similar biases can be calculated and displayed for the codons in the corresponding coding sequences.

*AAexplore* is equipped with a filter that allows the exclusion of polyQs that do not match either textual or numerical criteria. Analyses on e.g. polyQs with defined length ranges, defined GO tags or with OMIM annotations are possible. Finally, all the outputs can be exported as text data for further analyses with other applications.

### Availability

AAstretch package includes the *AAprepare*, *AAsync*, *AAstretch* and *AAexplore* programs. It is available as platform independent Perl scripts (tested on Linux, Mac OS X and Windows platforms). It can be downloaded from the the AAstrech Project website www.unifi.it/scibio/bioinfo/aastretch/ together with the manual and ready-to-use and yearly updated genomic preformatted files for most of the organisms available at the EnsEMBL database. The collection of polyQs generated for this work are also available (genomes from EnsEMBL release October, 2010)

### Statistics

The significance of the differences in residue or codon composition in polyQ zones compared to the whole genome composition was calculated using the chi-square statistics. The expected (*exp*) frequency of residue or codons was computed using two different background models: 1- sequences of all the proteins in the genome and 2- sequences of polyQ-containing proteins. In all cases the Q residue and its counts were excluded from the analysis resulting in 18 degrees of freedom. These frequencies were compared to the frequencies calculated in polyQ zones (*obs*) using the *X* ^2^ statistics:
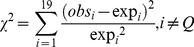
(6)For single residue analysis, we considered the case where the *obs*/*exp* ratios were higher than 2 or lower than 0.5, since a |log2 ratio|>1 is a widely accepted and statistically robust threshold, usually giving rise to significant (p<0.05) results when tested with X^2^ with one degree of freedom.

The distributions of the three parameters analyzed in Figure 3 (imperfect polyQs length, length of the longest pure polyQ for each polyQ zone and length of the longest pure polyCAG for each polyQ zone) were compared using the Mann-Whitney and Kolmogorov-Smirnov tests. Two distributions were considered significantly different when both tests were positive. The results are presented as box plots, in which a “box” comprises the two middle quartiles separated by the median, and the outliers are represented as open circles.

The available mammalian proteomes were checked for almost identical codon usage.

## Supporting Information

Figure S1 **Sequence biases at polyQ flanks are conserved throughout eukaryotic proteomes.** The relative abundances of Pro (A), His (B) and Asp (C) residues at polyQ flanks from the 15 eukaryotic proteomes we analyzed are shown. Black circles, Nt flanks; Gray circles, Ct flanks. The solid lines are the best fit to an exponential function. The dotted lines indicate the threshold for residues over- (residue twice as frequent as in the proteome) or under- (residue twice less frequent than in the proteome) representation.
(TIF)

Figure S2 **Non-homologous proteins exhibit similar sequence biases within polyQs.** The relative abundances of Pro (B), His (C), Leu (D), Asp (E), Cys (F) and Gly (G) residues within polyQs insertions are represented for all the polyQ-containing proteins (black) and for only the polyQ-containing proteins with no orthologous counterparts among the human polyQ-containing proteins (gray) in 6 different proteomes. The dotted lines indicate the threshold for residues over- (residue twice as frequent as in the proteome) or under- (residue twice less frequent than in the proteome) representation.
(TIF)

Figure S3 **GO tags associated to polyQs.** (A–C) GO tags associated to human polyQs: Biological process (A), Molecular function (B), Cellular component (C). (D) Proportion of polyQs tagged by “regulation of transcription DNA-dependent” (Biological process GO0006355) in different eukaryotic proteomes.
(TIF)

Figure S4 **Schematic representation of the strategy used by AAstretch.** *AAprepare* generates two organism specific input files containing fasta formatted coding sequences or their corresponding protein sequences from several databases available trough the EnsEMBL ftp site and the BioMart search engine. The two files are synchronized at the level of the fasta header, i.e. a coding sequence and its corresponding protein sequence share exactly the same fasta header containing several different annotations type. *AAstretch* analyzes the protein file following the user settings and polyXs and their flanking regions are retrieved. *AAstretch* generates a text-based output read by *AAsync* that uses the coding sequences prepared by *AAprepare* to overlay the polyX stretches and flanks into the corresponding coding sequences and generate a new output used for codon analysis. Both files are finally operated by *AAexplore* to generate a number of graphical representations for data analysis.
(TIF)

Table S1 **PolyQ-containing proteins for which polymorphism in the polyQ length has been reported as a risk or protective factor in multifactorial diseases.**
(RTF)

## References

[1] Chiti F, Dobson CM (2006). Protein misfolding, functional amyloid, and human disease.. Annu Rev Biochem.

[2] Hands SL, Wyttenbach A (2010). Neurotoxic protein oligomerisation associated with polyglutamine diseases.. Acta Neuropathol.

[3] Ross CA, Tabrizi SJ (2010). Huntington's disease: from molecular pathogenesis to clinical treatment.. Lancet Neurol.

[4] Zoghbi HY, Orr HT (2008). Pathogenic mechanisms of a polyglutamine-mediated neurodegenerative disease, spinocerebellar ataxia type 1.. J Biol Chem.

[5] Duyao M, Ambrose C, Myers R, Novelletto A, Persichetti F (1993). Trinucleotide repeat length instability and age of onset in Huntington's disease.. Nat Genet.

[6] Langbehn DR, Hayden MR, Paulsen JS (2009). CAG-repeat length and the age of onset in Huntington disease (HD): a review and validation study of statistical approaches.. Am J Med Genet B Neuropsychiatr Genet.

[7] Scherzinger E, Sittler A, Schweiger K, Heiser V, Lurz R (1999). Self-assembly of polyglutamine-containing huntingtin fragments into amyloid-like fibrils: implications for Huntington's disease pathology.. Proc Natl Acad Sci U S A.

[8] Rolfsmeier ML, Lahue RS (1999). Stabilizing effects of interruptions on trinucleotide repeat expansions in Saccharomyces cerevisiae.. Mol Cell Biol.

[9] Sobczak K, Krzyzosiak WJ (2004). Patterns of CAG repeat interruptions in SCA1 and SCA2 genes in relation to repeat instability.. Hum Mutat.

[10] Wexler NS, Lorimer J, Porter J, Gomez F, Moskowitz C (2004). Venezuelan kindreds reveal that genetic and environmental factors modulate Huntington's disease age of onset.. Proc Natl Acad Sci U S A.

[11] Scherzinger E, Lurz R, Turmaine M, Mangiarini L, Hollenbach B (1997). Huntingtin-encoded polyglutamine expansions form amyloid-like protein aggregates in vitro and in vivo.. Cell.

[12] de Chiara C, Menon RP, Dal Piaz F, Calder L, Pastore A (2005). Polyglutamine is not all: the functional role of the AXH domain in the ataxin-1 protein.. J Mol Biol.

[13] Harris GM, Dodelzon K, Gong L, Gonzalez-Alegre P, Paulson HL (2010). Splice isoforms of the polyglutamine disease protein ataxin-3 exhibit similar enzymatic yet different aggregation properties.. PLoS ONE.

[14] Menon RP, Pastore A (2006). Expansion of amino acid homo-sequences in proteins: insights into the role of amino acid homo-polymers and of the protein context in aggregation.. Cell Mol Life Sci.

[15] Paulson HL, Perez MK, Trottier Y, Trojanowski JQ, Subramony SH (1997). Intranuclear inclusions of expanded polyglutamine protein in spinocerebellar ataxia type 3.. Neuron.

[16] Qin ZH, Wang Y, Sapp E, Cuiffo B, Wanker E (2004). Huntingtin bodies sequester vesicle-associated proteins by a polyproline-dependent interaction.. J Neurosci.

[17] Bhattacharyya A, Thakur AK, Chellgren VM, Thiagarajan G, Williams AD (2005). Oligoproline effects on polyglutamine conformation and aggregation.. J Mol Biol.

[18] Darnell G, Orgel JP, Pahl R, Meredith SC (2007). Flanking polyproline sequences inhibit beta-sheet structure in polyglutamine segments by inducing PPII-like helix structure.. J Mol Biol.

[19] Dehay B, Bertolotti A (2006). Critical role of the proline-rich region in Huntingtin for aggregation and cytotoxicity in yeast.. J Biol Chem.

[20] Duennwald ML, Jagadish S, Muchowski PJ, Lindquist S (2006). Flanking sequences profoundly alter polyglutamine toxicity in yeast.. Proc Natl Acad Sci U S A.

[21] Rockabrand E, Slepko N, Pantalone A, Nukala VN, Kazantsev A (2006). The first 17 amino acids of Huntingtin modulate its sub-cellular localization, aggregation and effects on calcium homeostasis.. Hum Mol Genet.

[22] Tam S, Spiess C, Auyeung W, Joachimiak L, Chen B (2009). The chaperonin TRiC blocks a huntingtin sequence element that promotes the conformational switch to aggregation.. Nat Struct Mol Biol.

[23] Thakur AK, Jayaraman M, Mishra R, Thakur M, Chellgren VM (2009). Polyglutamine disruption of the huntingtin exon 1 N terminus triggers a complex aggregation mechanism.. Nat Struct Mol Biol.

[24] Huntley MA, Clark AG (2007). Evolutionary analysis of amino acid repeats across the genomes of 12 Drosophila species.. Mol Biol Evol.

[25] Chung MY, Ranum LP, Duvick LA, Servadio A, Zoghbi HY, Orr HT (1993). Evidence for a mechanism predisposing to intergenerational CAG repeat instability in spinocerebellar ataxia type I.. Nat Genet.

[26] Karlin S (1995). Statistical significance of sequence patterns in proteins.. Curr Opin Struct Biol.

[27] Faux NG, Bottomley SP, Lesk AM, Irving JA, Morrison JR (2005). Functional insights from the distribution and role of homopeptide repeat-containing proteins.. Genome Res.

[28] Karlin S, Brocchieri L, Bergman A, Mrazek J, Gentles AJ (2002). Amino acid runs in eukaryotic proteomes and disease associations.. Proc Natl Acad Sci U S A.

[29] Siwach P, Pophaly SD, Ganesh S (2006). Genomic and evolutionary insights into genes encoding proteins with single amino acid repeats.. Mol Biol Evol.

[30] Rebbeck TR, Wang Y, Kantoff PW, Krithivas K, Neuhausen SL (2001). Modification of BRCA1- and BRCA2-associated breast cancer risk by AIB1 genotype and reproductive history.. Cancer Res.

[31] Wong LJ, Dai P, Lu JF, Lou MA, Clarke R, Nazarov V (2006). AIB1 gene amplification and the instability of polyQ encoding sequence in breast cancer cell lines.. BMC Cancer.

[32] Kumar R, Atamna H, Zakharov MN, Bhasin S, Khan SH, Jasuja R (2011). Role of the androgen receptor CAG repeat polymorphism in prostate cancer, and spinal and bulbar muscular atrophy.. Life Sci.

[33] Marcotte EM, Pellegrini M, Yeates TO, Eisenberg D (1999). A census of protein repeats.. J Mol Biol.

[34] Faux NG, Huttley GA, Mahmood K, Webb GI, de la Banda MG, Whisstock JC (2007). RCPdb: An evolutionary classification and codon usage database for repeat-containing proteins.. Genome Res.

[35] Mularoni L, Veitia RA, Albà MM (2007). Highly constrained proteins contain an unexpectedly large number of amino acid tandem repeats.. Genomics.

[36] Albà MM, Guigó R (2004). Comparative analysis of amino acid repeats in rodents and humans.. Genome Res.

[37] Katti MV, Sami-Subbu R, Ranjekar PK, Gupta VS (2000). Amino acid repeat patterns in protein sequences: their diversity and structural-functional implications.. Protein Sci.

[38] Kozlowski P, de Mezer M, Krzyzosiak WJ (2010). Trinucleotide repeats in human genome and exome.. Nucleic Acids Res.

[39] Simon M, Hancock JM (2009). Tandem and cryptic amino acid repeats accumulate in disordered regions of proteins.. Genome Biol.

[40] Garvie CW, Wolberger C (2001). Recognition of specific DNA sequences.. Mol Cell.

[41] Miller M (2009). The importance of being flexible: the case of basic region leucine zipper transcriptional regulators.. Curr Protein Pept Sci.

[42] Haerty W, Golding GB (2010). Genome-wide evidence for selection acting on single amino acid repeats.. Genome Res.

[43] Gojobori J, Ueda S (2010). Elevated evolutionary rate in genes with homopolymeric amino acid repeats constituting nondisordered structure.. Mol Biol Evol.

[44] Huang H, Winter EE, Wang H, Weinstock KG, Xing H (2004). Evolutionary conservation and selection of human disease gene orthologs in the rat and mouse genomes.. Genome Biol.

[45] Tartari M, Gissi C, Lo Sardo V, Zuccato C, Picardi E (2007). Phylogenetic comparison of huntingtin homologues reveals the appearance of a primitive polyQ in sea urchin.. Mol Biol Evol.

[46] Albà MM, Santibáñez-Koref MF, Hancock JM (2001). The comparative genomics of polyglutamine repeats: extreme differences in the codon organization of repeat-encoding regions between mammals and Drosophila.. J Mol Evol.

[47] Madsen LB, Thomsen B, Sølvsten CA, Bendixen C, Fredholm M (2007). Identification of the porcine homologous of human disease causing trinucleotide repeat sequences.. Neurogenetics.

[48] Kurosaki T, Ninokata A, Wang L, Ueda S (2006). Evolutionary scenario for acquisition of CAG repeats in human SCA1 gene.. Gene.

[49] Pearson CE, Nichol Edamura K, Cleary JD (2005). Repeat instability: mechanisms of dynamic mutations.. Nat Rev Genet.

[50] Sobczak K, Krzyzosiak WJ (2004). CAG repeats containing CAA interruptions form branched hairpin structures in spinocerebellar ataxia type 2 transcripts.. J Biol Chem.

[51] Eichler EE, Holden JJ, Popovich BW, Reiss AL, Snow K (1994). Length of uninterrupted CGG repeats determines instability in the FMR1 gene.. Nat Genet.

[52] Sakamoto N, Larson JE, Iyer RR, Montermini L, Pandolfo M, Wells RD (2001). GGA*TCC-interrupted triplets in long GAA*TTC repeats inhibit the formation of triplex and sticky DNA structures, alleviate transcription inhibition, and reduce genetic instabilities.. J Biol Chem.

[53] Dixon MJ, Lahue RS (2004). DNA elements important for CAG*CTG repeat thresholds in Saccharomyces cerevisiae.. Nucleic Acids Res.

[54] Hsu RJ, Hsiao KM, Lin MJ, Li CY, Wang LC (2011). Long tract of untranslated CAG repeats is deleterious in transgenic mice.. PLoS ONE.

[55] Li LB, Yu Z, Teng X, Bonini NM (2008). RNA toxicity is a component of ataxin-3 degeneration in Drosophila.. Nature.

[56] Wang LC, Chen KY, Pan H, Wu CC, Chen PH (2010). Muscleblind participates in RNA toxicity of expanded CAG and CUG repeats in Caenorhabditis elegans.. Cell Mol Life Sci.

[57] de Mezer M, Wojciechowska M, Napierala M, Sobczak K, Krzyzosiak WJ (2011). Mutant CAG repeats of Huntingtin transcript fold into hairpins, form nuclear foci and are targets for RNA interference.. Nucleic Acids Res.

[58] Kiliszek A, Kierzek R, Krzyzosiak WJ, Rypniewski W (2010). Atomic resolution structure of CAG RNA repeats: structural insights and implications for the trinucleotide repeat expansion diseases.. Nucleic Acids Res.

[59] Sobczak K, Michlewski G, de Mezer M, Kierzek E, Krol J (2010). Structural diversity of triplet repeat RNAs.. J Biol Chem.

[60] Todd PK, Paulson HL (2010). RNA-mediated neurodegeneration in repeat expansion disorders.. Ann Neurol.

[61] Lawlor KT, O'Keefe LV, Samaraweera SE, van Eyk CL, McLeod CJ (2011). Double-stranded RNA is pathogenic in Drosophila models of expanded repeat neurodegenerative diseases..

[62] Jasinska A, Michlewski G, de Mezer M, Sobczak K, Kozlowski P (2003). Structures of trinucleotide repeats in human transcripts and their functional implications.. Nucleic Acids Res.

[63] Michlewski G, Krzyzosiak WJ (2004). Molecular architecture of CAG repeats in human disease related transcripts.. J Mol Biol.

[64] Sobczak K, Krzyzosiak WJ (2004). Imperfect CAG repeats form diverse structures in SCA1 transcripts.. J Biol Chem.

[65] Bauer PO, Nukina N (2009). The pathogenic mechanisms of polyglutamine diseases and current therapeutic strategies.. J Neurochem.

[66] Wang Y, Meriin AB, Zaarur N, Romanova NV, Chernoff YO (2008). Abnormal proteins can form aggresome in yeast: aggresome-targeting signals and components of the machinery.. FASEB J.

[67] Popiel HA, Nagai Y, Onodera O, Inui T, Fujikake N (2004). Disruption of the toxic conformation of the expanded polyglutamine stretch leads to suppression of aggregate formation and cytotoxicity.. Biochem Biophys Res Commun.

[68] Sen S, Dash D, Pasha S, Brahmachari SK (2003). Role of histidine interruption in mitigating the pathological effects of long polyglutamine stretches in SCA1: A molecular approach.. Protein Sci.

[69] Lakhani VV, Ding F, Dokholyan NV (2010). Polyglutamine induced misfolding of huntingtin exon1 is modulated by the flanking sequences.. PLoS Comput Biol.

[70] Jayaraman M, Kodali R, Wetzel R (2009). The impact of ataxin-1-like histidine insertions on polyglutamine aggregation.. Protein Eng Des Sel.

[71] Sharma D, Sharma S, Pasha S, Brahmachari SK (1999). Peptide models for inherited neurodegenerative disorders: conformation and aggregation properties of long polyglutamine peptides with and without interruptions.. FEBS Lett.

[72] Rousseau F, Serrano L, Schymkowitz JW (2005). How evolutionary pressure against protein aggregation shaped chaperone specificity.. J Mol Biol.

[73] Reumers J, Maurer-Stroh S, Schymkowitz J, Rousseau F (2009). Protein sequences encode safeguards against aggregation.. Hum Mutat.

[74] Monsellier E, Chiti F (2007). Prevention of amyloid-like aggregation as a driving force of protein evolution.. EMBO Rep.

[75] Ashburner M, Ball CA, Blake JA, Botstein D, Butler H (2000). Gene ontology: tool for the unification of biology. The Gene Ontology Consortium.. Nat Genet.

[76] McKusick VA (2007). Mendelian Inheritance in Man and its online version, OMIM.. Am J Hum Genet.

[77] Hubbard TJ, Aken BL, Ayling S, Ballester B, Beal K (2008). Ensembl 2009.. Nucleic Acids Res.

[78] Kersey PJ, Lawson D, Birney E, Derwent PS, Haimel M (2009). Ensembl Genomes: extending Ensembl across the taxonomic space.. Nucleic Acids Res.

[79] Haider S, Ballester B, Smedley D, Zhang J, Rice P, Kasprzyk A (2009). BioMart Central Portal–unified access to biological data.. Nucleic Acids Res.

[80] Tatusov RL, Fedorova ND, Jackson JD, Jacobs AR, Kiryutin B (2003). The COG database: an updated version includes eukaryotes.. BMC Bioinformatics.

