# Supplementary figures and images for: Polyglutamine Repeats Are Associated to Specific Sequence Biases That Are Conserved among Eukaryotes
Source: PLoS One. 2012 Feb 1;7(2):e30824. doi: 10.1371/journal.pone.0030824 (PMC3270027; doi:10.1371/journal.pone.0030824)

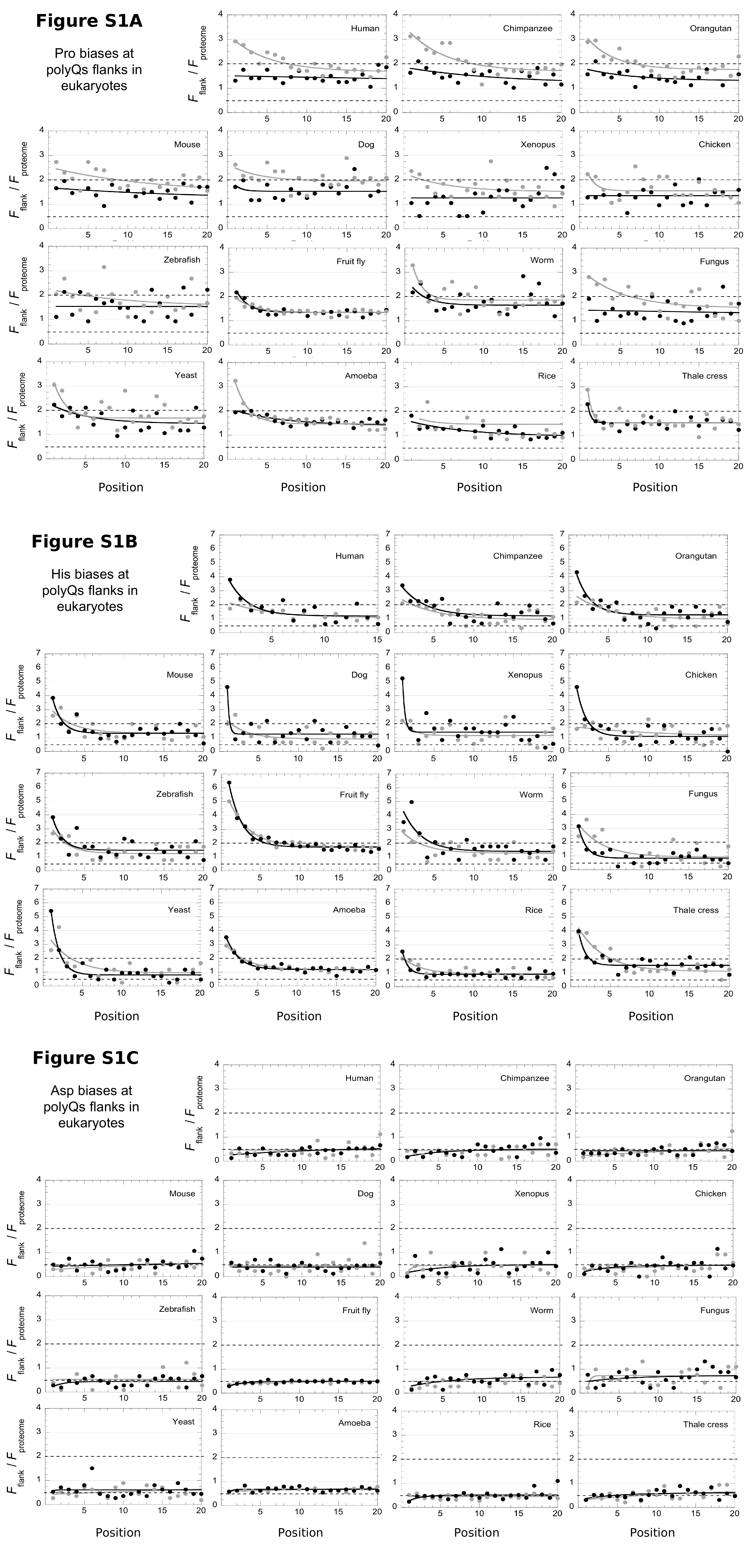

Supplement: Figure S1 — Sequence biases at polyQ flanks are conserved throughout eukaryotic proteomes. The relative abundances of Pro (A), His (B) and Asp (C) residues at polyQ flanks from the 15 eukaryotic proteomes we analyzed are shown. Black circles, Nt flanks; Gray circles, Ct flanks. The solid lines are the best fit to an exponential function. The dotted lines indicate the threshold for residues over- (residue twice as frequent as in the proteome) or under- (residue twice less frequent than in the proteome) representation. (TIF) [file pone.0030824.s001.tif]

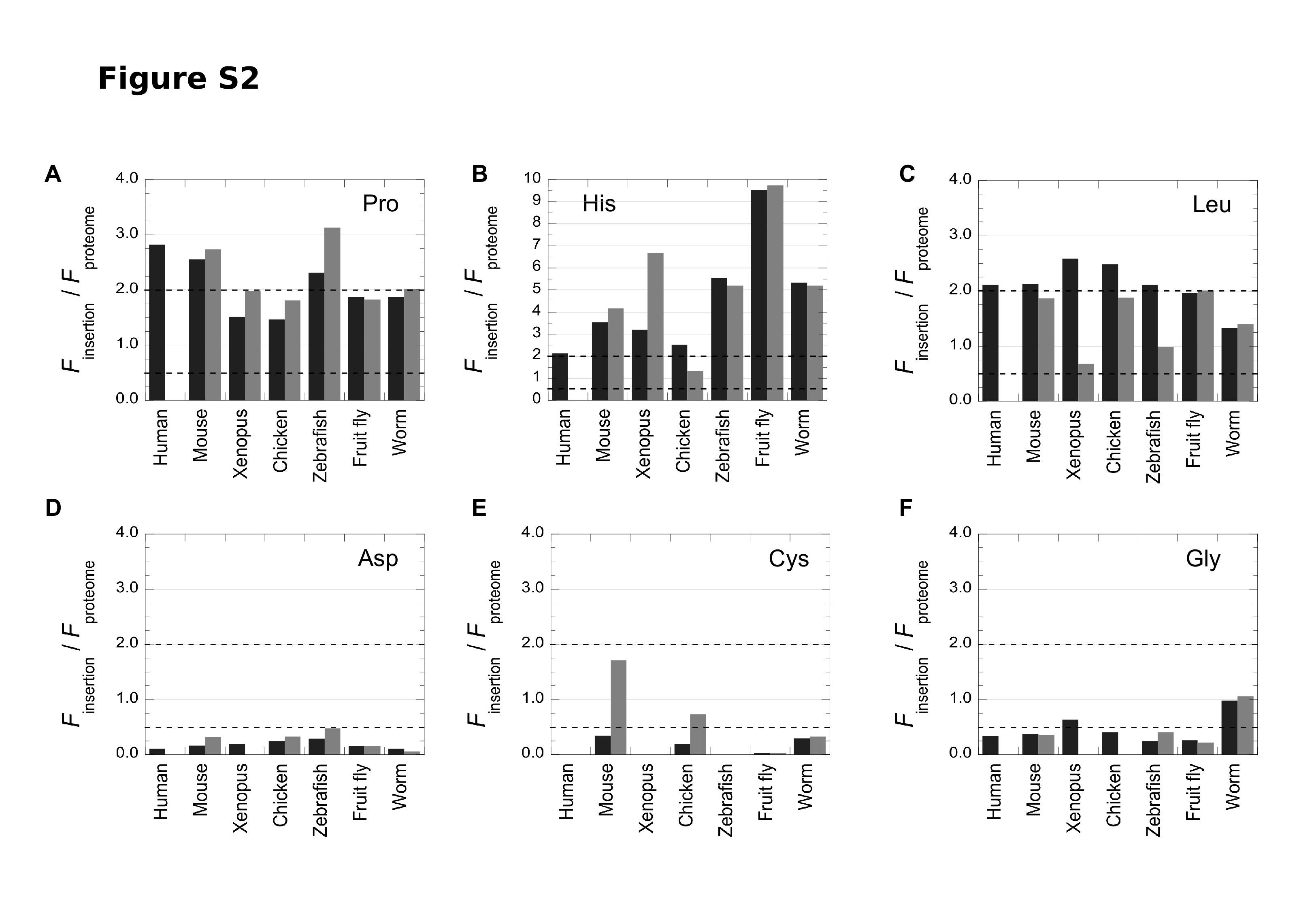

Supplement: Figure S2 — Non-homologous proteins exhibit similar sequence biases within polyQs. The relative abundances of Pro (B), His (C), Leu (D), Asp (E), Cys (F) and Gly (G) residues within polyQs insertions are represented for all the polyQ-containing proteins (black) and for only the polyQ-containing proteins with no orthologous counterparts among the human polyQ-containing proteins (gray) in 6 different proteomes. The dotted lines indicate the threshold for residues over- (residue twice as frequent as in the proteome) or under- (residue twice less frequent than in the proteome) representation. (TIF) [file pone.0030824.s002.tif]

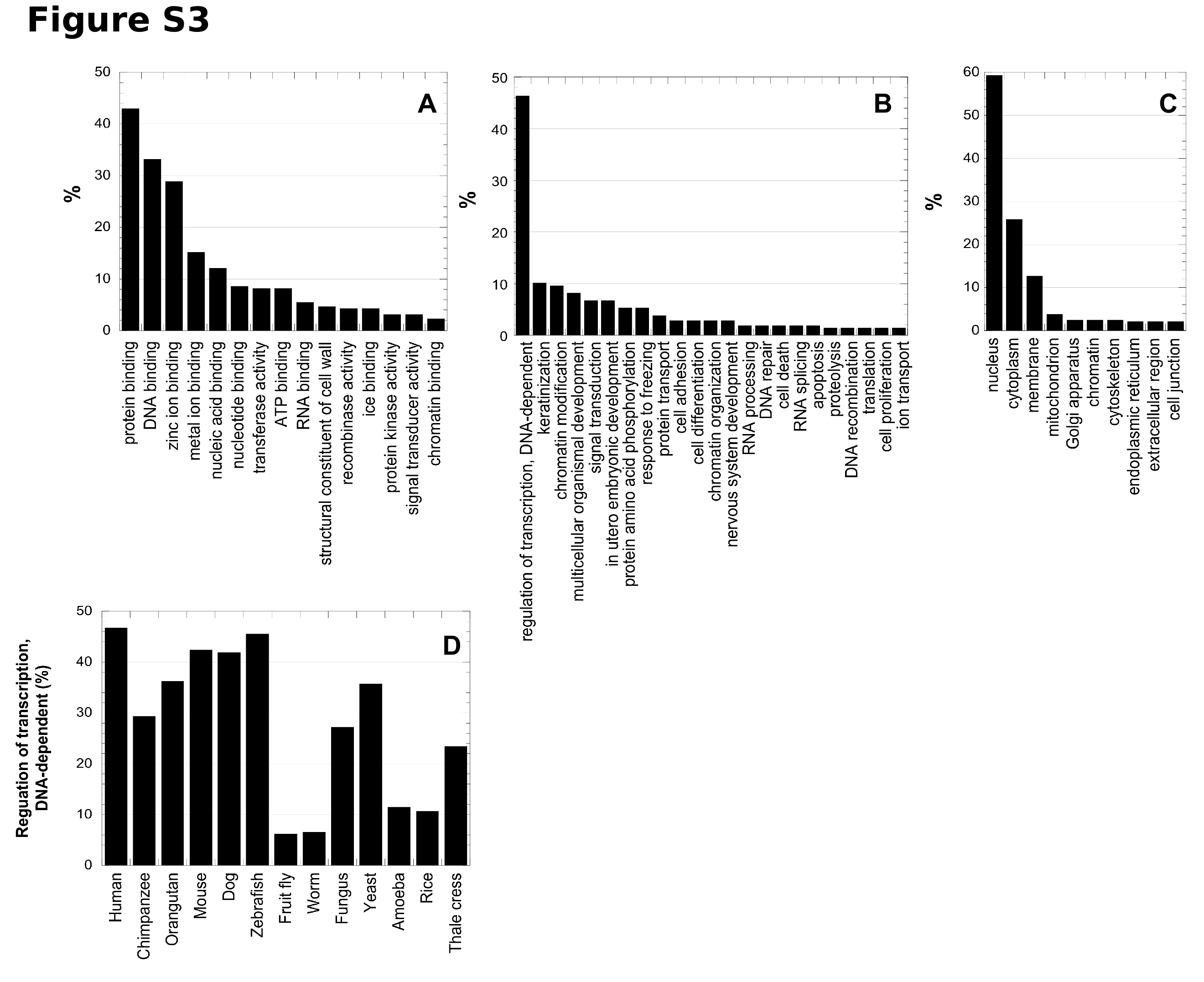

Supplement: Figure S3 — GO tags associated to polyQs. (A–C) GO tags associated to human polyQs: Biological process (A), Molecular function (B), Cellular component (C). (D) Proportion of polyQs tagged by “regulation of transcription DNA-dependent” (Biological process GO0006355) in different eukaryotic proteomes. (TIF) [file pone.0030824.s003.tif]

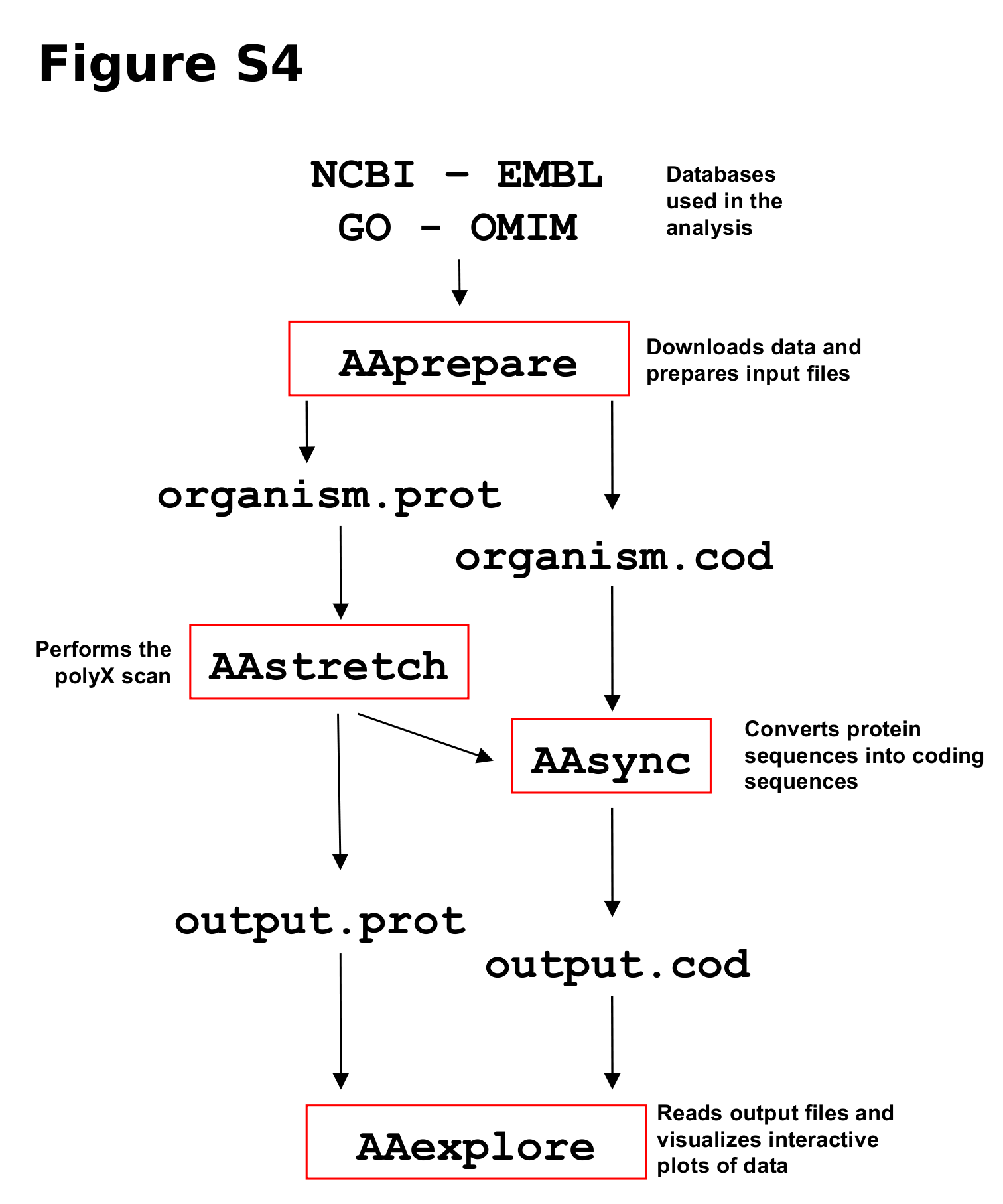

Supplement: Figure S4 — Schematic representation of the strategy used by AAstretch. AAprepare generates two organism specific input files containing fasta formatted coding sequences or their corresponding protein sequences from several databases available trough the EnsEMBL ftp site and the BioMart search engine. The two files are synchronized at the level of the fasta header, i.e. a coding sequence and its corresponding protein sequence share exactly the same fasta header containing several different annotations type. AAstretch analyzes the protein file following the user settings and polyXs and their flanking regions are retrieved. AAstretch generates a text-based output read by AAsync that uses the coding sequences prepared by AAprepare to overlay the polyX stretches and flanks into the corresponding coding sequences and generate a new output used for codon analysis. Both files are finally operated by AAexplore to generate a number of graphical representations for data analysis. (TIF) [file pone.0030824.s004.tif]
